# Preparation of Doxorubicin-Loaded Amphiphilic Poly(D,L-Lactide-*Co*-Glycolide)-b-Poly(*N*-Acryloylmorpholine) AB_2_ Miktoarm Star Block Copolymers for Anticancer Drug Delivery

**DOI:** 10.3390/ma13173713

**Published:** 2020-08-22

**Authors:** Kalyan Ramesh, Avnish Kumar Mishra, Jin Kon Kim, Yeon Tae Jeong, Yeong-Soon Gal, Kwon Taek Lim

**Affiliations:** 1 Department of Display Engineering, Pukyong National University, Busan 48513, Korea; kramesh.chem@gmail.com (K.R.); ytjeong@pknu.ac.kr (Y.T.J.); 2 National Creative Research Initiative Center for Smart Block Copolymers, Department of Chemical Engineering, Pohang University of Science and Technology, Pohang 37673, Korea; avnishbhu@gmail.com (A.K.M.); jkkim@postech.ac.kr (J.K.K.); 3 Department of Fire Safety, Kyungil University, Gyeongsan 34828, Korea; ysgal@kiu.ac.kr

**Keywords:** miktoarm amphiphilic block copolymers, drug delivery, RAFT polymerization, ROP polymerization

## Abstract

Owing to their unique topology and physical properties, micelles based on miktoarm amphiphilic star block copolymers play an important role in the biomedical field for drug delivery. Herein, we developed a series of AB_2_-type poly(D,L-lactide-co-glycolide)-b-poly(*N*-acryloyl morpholine) (PLGA-b-PNAM_2_) miktoarm star block copolymers by reversible addition–fragmentation chain–transfer polymerization and ring-opening copolymerization. The resulting miktoarm star polymers were investigated by ^1^H NMR spectroscopy and gel permeation chromatography. The critical micellar concentration value of the micelles increases with an increase in PNAM block length. As revealed by transmission electron microscopy and dynamic light scattering, the amphiphilic miktoarm star block copolymers can self-assemble to form spherical micellar aggregates in water. The anticancer drug doxorubicin (DOX) was encapsulated by polymeric micelles; the drug-loading efficiency and drug-loading content of the DOX-loaded micelles were 81.7% and 9.1%, respectively. Acidic environments triggered the dissociation of the polymeric micelles, which led to the more release of DOX in pH 6.4 than pH 7.4. The amphiphilic PLGA-b-PNAM_2_ miktoarm star block copolymers may have broad application as nanocarriers for controlled drug delivery.

## 1. Introduction

In the current decade, polymeric micelles based on amphiphilic block copolymers (ABCs) have been broadly explored as nanocarriers in the drug-delivery field due to their advantages such as long circulation, molecular design, therapeutic effect and biocompatibility. In addition, the ability of ABCs to self-assemble into complex structures has allowed improved drug loading [1,2]. The hydrophobic core of ABCs offers great potential to load hydrophobic drugs and controlled drug release through micellar dissociation, polymer erosion/degradation or diffusion mechanisms [3,4]. Among ABCs, the miktoarm star copolymers synthesized with varying polymer arms and molecular weights have been attractive owing to their interesting properties [5,6,7]. Miktoarm polymers contain two or more polymeric units with various chemical structures. The miktoarm-block copolymers demonstrate exclusive phase-separation behavior either in solution or in bulk compared with linear-block copolymers due to the organization of the polymers, in which two constructional blocks are connected to a distinct confluence point [8,9,10,11]. This ability imparts miktoarm-block copolymers with wide potentials for use as nanocarriers of drugs compared with corresponding linear-block copolymers [12,13,14,15,16]. The synthesis of AB_2_ miktoarm-block copolymers is mostly practicable with a classification of different polymerization methods. To date, few reports have been published for the preparation of AB_2_-type miktoarm-block copolymers and their drug-delivery application [10,17,18,19,20,21]. Chong et al. have reported the AB_2_-type miktoarm star polymers containing monomethoxy poly(ethylene glycol) (mPEG) and poly(D,L-lactide-co-glycolide) (PLGA) blocks and the general advantage of these miktoarm-block copolymers as nanocarriers for ibuprofen [10]. Yoon et al. reported PEG–PCL_2_, and they investigated the self-aggregated structure, in vitro drug release using DOX and antitumor activities [17]. Soliman et al. also demonstrated the PEG–PCL_2_ miktoarm polymers for the nimodipine (NIM) drug release and these NIM-loaded miktoarm polymers were evaluated against murine microglia cell line in vitro for inflammation [18]. Yin et al. developed PEG–poly(L-lactide)_2_ miktoarm block copolymers. These polymersomes demonstrated the potent drug-loading capacities of DOX and showed significant drug release after 48 h [19]. Recently, we have prepared a AB_2_-type of miktoarm-block copolymer composed of poly(N-acryloylmorpholine) and poly(D,L-lactide) as a nano carrier for drug delivery [22]. PLGA is a generally explored drug carrier for the treatment of several diseases and some invention products of PLGA are currently used in clinical trial [1,23]. The key attraction for further exploitation of PLGA is its biodegradability and biocompatibility, along with good bioresorptivity of its degradation products [24,25]. However, its capability in drug-release application is limited by the high hydrophobicity of PLGA [23,26,27]. Poly(*N*-acryloylmorpholine) (PNAM)—a water-soluble polymer—is an acrylamide derivative with a heterocyclic tetrahydrooxazine substituent [28,29,30]. PNAM is one of promising polymers, similarly advantageous as PEG for biologic applications due to its solubility in various organic solvents and biocompatibility [31,32]. Because of these properties, they find numerous applications in the biomedical field. They are also utilized in chromatography, strong-stage combination of peptides, catalysis and arrangement of composite semi-penetrable layers [33,34,35,36]. Considering the advantages of both PLGA and PNAM polymers, a combination of the polymers to make a miktoarm-block copolymer could offer more promising results that can be used for drug-delivery applications. Herein, we first report a miktoarm star block copolymer consisting of PLGA hydrophobic blocks and PNAM hydrophilic blocks, prepared from the reversible addition–fragmentation chain–transfer (RAFT) method. The miktoarm star block copolymers could self-aggregate to form spherical micellar aggregates in water, which were investigated by dynamic light scattering (DLS) and transmission electron microscopy (TEM). An anticancer drug, DOX was efficiently encapsulated into micelles, and the DOX-loaded micelles exhibited sustained controlled drug-delivery properties.

## 2. Results and Discussion

### 2.1. Synthesis of PLGA-b-PNAM_2,_ the AB_2_-Type Miktoarm Star Block Copolymers

**Scheme 1:**
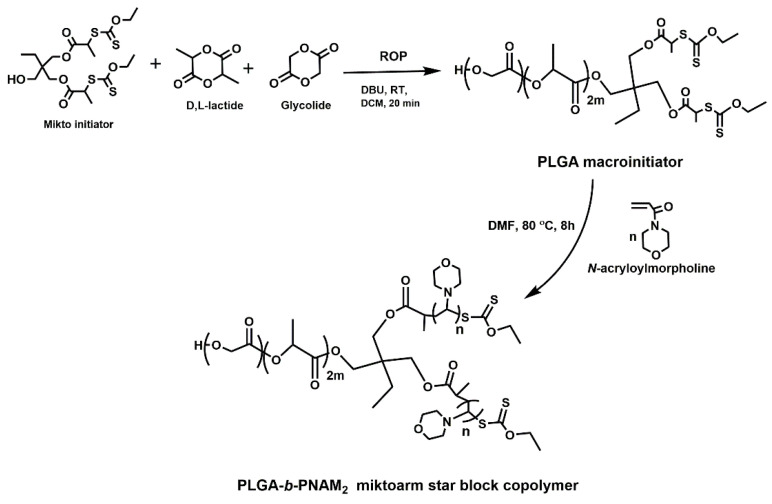
Synthetic scheme for poly(D,L-lactide-co-glycolide)-b-poly(*N*-acryloyl morpholine) (PLGA-b-PNAM_2_) miktoarm star block copolymers.

**Figure 1:**
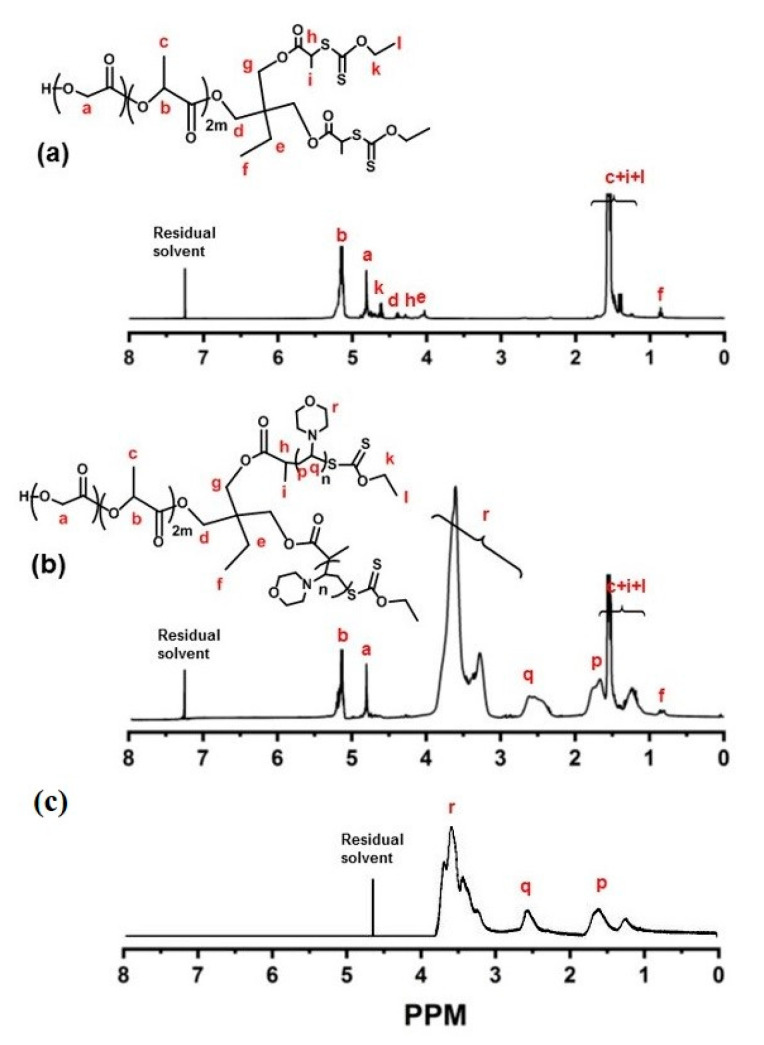
^1^H NMR spectra of (**a**) the poly(D,L-lactide-co-glycolide) (PLGA) macroinitiator and PLGA-b-PNAM_2_ miktoarm-block copolymer (M2) in (**b**) CDCl_3_ and (**c**) D_2_O at room temperature.

**Figure 2:**
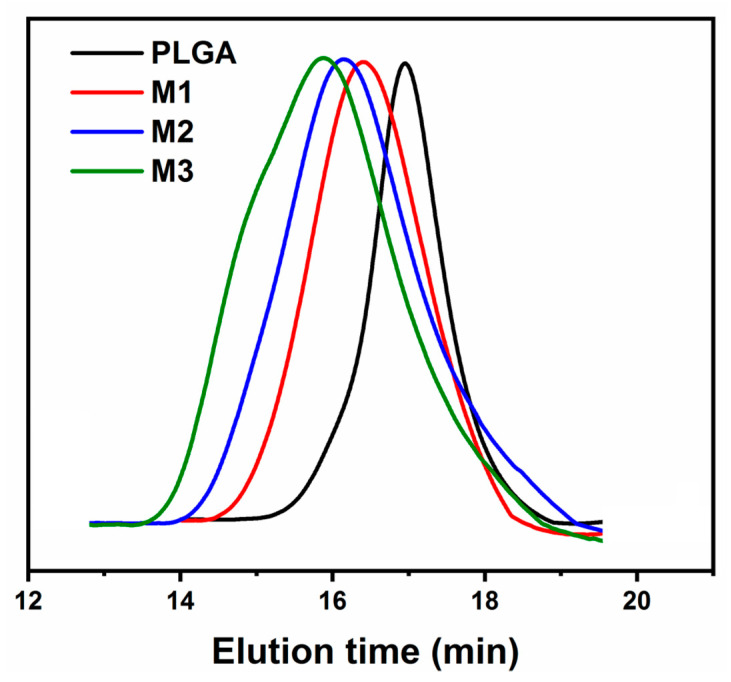
Gel permeation chromatography (GPC) curve of the PLGA macroinitiator and the miktoarm PLGA-b-PNAM_2_ star block copolymer (Table 1).

The three miktoarm amphiphilic star block copolymers of PLGA-b-PNAM_2_ were prepared by the two-step method which consisted of the synthesis of PLGA macroinitiators by the ring-opening polymerization (ROP) of glycolide and D,L-lactide, followed by the RAFT polymerization of NAM (Scheme 1). In the first step, the ring opening copolymerization of glycolide and D,L-lactide (70/30, *w/w*) was carried out in the presence of the miktoarm initiator using 1,8-diazabicyclo [5.4.0] undec-7-ene (DBU) as a catalyst at room temperature. The ^1^H NMR spectra of the PLGA macro initiator is presented in Figure 1a. The signals at 4.88–4.78 ppm, 5.25–5.12 and 1.59–1.52 ppm correspond to the PLGA and poly(D,L-lactic acid) (PDLLA) backbones, whereas the signal at 4.64–4.62 ppm corresponds to the xanthate functional group of the miktoarm initiator. Therefore, ^1^H NMR investigation confirms that the PLGA macroinitiator was successfully prepared. The integrated peak areas “b” of the PDLLA backbone chain and ‘‘a’’ of the PGA backbone chain were compared by the integrated peak area of ‘‘k’’ of the chain-end xanthate moiety of the miktoarm initiator, to estimate the M_n_ (NMR) of the PLGA copolymer, which was calculated to be 4800 g mol^−1^. The gel permeation chromatography (GPC) study of the copolymer specified that the molecular weight (M_n_) and dispersity (Đ) were 5300 g mol^−1^ and 1.20 (Figure 2). In the next step, miktoarm PLGA-b-PNAM_2_ star block copolymers were prepared through RAFT polymerization of NAM monomers with the PLGA macroinitiator at 80 °C for 8 h.

The PLGA-b-PNAM_2_ miktoarm star block copolymers with different PNAM chain lengths were synthesized by changing the feed ratios of the PLGA macroinitiator to NAM. These miktoarm star block copolymers are denominated M1, M2 and M3. The successful synthesis of the miktoarm star block copolymer was revealed by the ^1^H NMR spectrum. As shown in Figure 1b, new proton peaks are observed at 2.3–2.8 (*q*), 3.0–4.0 (*r*) and 1.70–1.90 (*p*) ppm, due to the presence of PNAM backbone chains. The GPC chromatogram shows that the elution times of the three miktoarm-block copolymers are shorter than that of the PLGA macroinitiator as can be seen in Figure 2, which indicates the formation of the desired miktoarm-block copolymers. The decrease in the retention time in order of M1, M2 and M3 suggests that the molecular weights of the miktoarm star block copolymers increase as the feed ratios of NAM monomers increase (Table 1) [37].

It is also noticeable that the GPC curves of M3 display shoulders to higher molecular weights while the shapes of M1 and M2 are unimodal. The bimodal-like shape of M3 may be attributed to the star–star coupling of the propagating species of miktoarm copolymers at the high ratio of NAM with respect to the macroinitiator [38]. In other words, the R–RAFT-type PLGA macroinitiator may leads to star–star coupling via termination of two star polymers carrying a radical center [39]. With the PLGA RAFT agents, two types of active propagating species, ones that are attached to the PLGA core chain and linear chains resulting from continuous initiation, can coexist. Radical-radical termination resulting from these species can thus produce star–star coupled polymers, star polymers with dead arms and linear dead polymers, which becomes pronounced at low concentrations of RAFT agent with the polymerization carried to high conversion [38].

### 2.2. Self-Assembly Study of Miktoarm PLGA-b-PNAM_2_ Amphiphilic Star Block Copolymers

**Figure 3:**
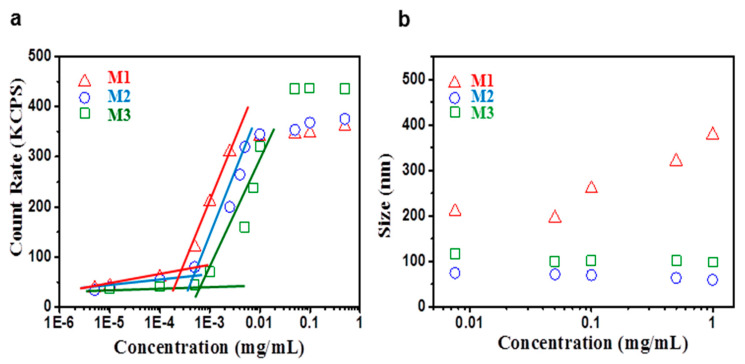
(**a**) Plot of the counter rate versus the concentration of M1, M2 and M3 in water; (**b**) plot of the micellar size in diameter versus the concentration of M1, M2 and M3 in water.

PLGA is insoluble in water, while PNAM is quite soluble in water. In Figure 1c, the ^1^HNMR spectrum of M2 in D_2_O clearly shows the appearance of PNAM and the peaks attributed to PLGA are suppressed in comparison with the ^1^H NMR spectrum obtained in chloroform-d which dissolves both polymers (Figure 1b). This result indicates that PLGA-b-PNAM_2_ miktoarm star block copolymers form micelles in water where the core and the shell consist of PLGA and PNAM chains, respectively. Figure 3a, represents the plot of the count rate of the micelles versus the concentration of M1, M2 and M3 in water, measured by DLS. Because a sudden increase in the count rate indicates the formation of micelle at critical micellar concentration (CMC), CMCs were determined as ~3.08 × 10^−4^, ~4.24 × 10^−4^ and ~7.90 × 10^−4^ mg/mL for M1, M2 and M3, respectively. These values indicate that the CMC of the amphiphilic miktoarm-block copolymers increases with the increase in the chain length of the PNAM block [24].

Figure 3b shows the plot of the size of the micelles (in diameter) versus concentration above the CMC value of M1, M2 and M3 in water, measured by DLS. We observed that above the CMC value the size of the micelle is stable in case of M2 and M3 whereas for M1 it changes. Further, M2 has a low CMC value compared with that of M3. Because of this, M2 was selected as the best candidate for further studies. We also measured the CMC and size of the micelles in various concentrations above the CMC in PBS (pH = 7.4) at 37 °C. The CMC values of M1, M2 and M3 in PBS are ~3.62 × 10^−4^, ~6.24 × 10^−4^ and ~8.50 × 10^−4^ mg/mL, respectively. These CMC values in PBS are slightly higher than water and the size of the micelles are also stable in PBS (pH = 7.4) at various concentration (Figure S1, see supporting information). The stability of the micelles shows their potential as a drug nanocarrier in biological applications. [40].

**Figure 4:**
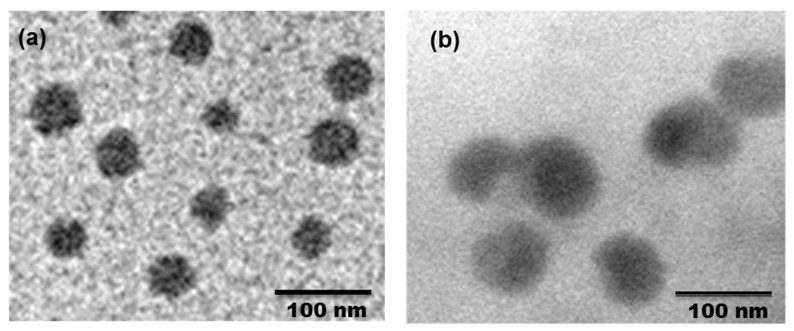
TEM study of (**a**) blank micelles and (**b**) doxorubicin (DOX)-loaded micelles of M2.

**Figure 5:**
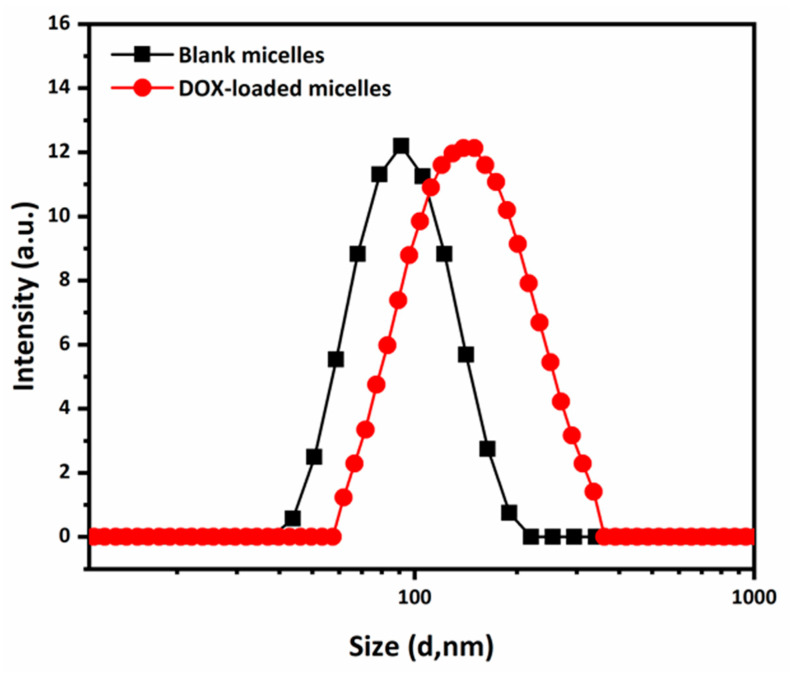
Dynamic light scattering (DLS) study of (**a**) blank micelles and (**b**) DOX-loaded micelles of M2.

In Figure 4a, the TEM measurement of M2 micelles showed spherical shapes with an average diameter of 47.5 nm. The hydrodynamic diameter of the M2 micelles was also examined by DLS and the average diameter was 68.7 nm (Figure 5). The average diameter of the micelles is somewhat larger than a theoretical value which may be calculated from a model of chain folding based on the chain length and molecular weight. This can be attributed to the poorly defined structure of the block copolymers. However, the size of micellar aggregates is belong to the suitable size range (40–200 nm) of the enhanced permeability and retention(EPR) effect, while the size is large enough to preclude fast renal clearance [41,42]. Longer circulation allows the micelles to accumulate to a greater extent in areas with a defective or leaky vasculature such as tumors via the EPR effect. Therefore, we investigated the in vitro drug loading and release study using the M2 micellar aggregates.

### 2.3. In Vitro Drug Loading and Release Study

**Figure 6:**
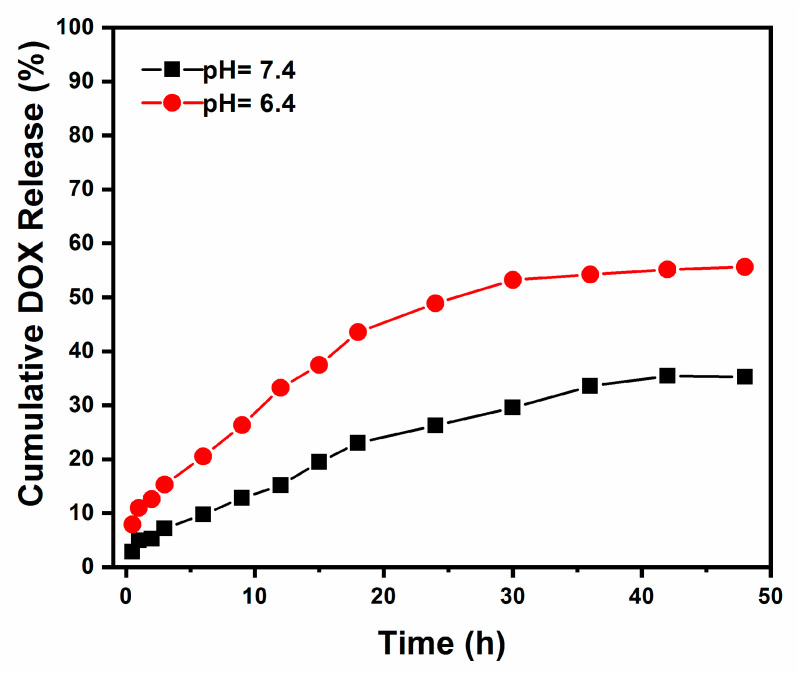
Drug-release profile of DOX-loaded M2 in PBS (pH = 7.4/6.4) at 37 °C.

DOX was incorporated into the core of M2 micelles using the dialysis system. The average diameter of the drug-loaded micelles measured to be 71.2 nm by TEM (Figure 4b) and 112.3 nm by DLS (Figure 5). The results revealed that the size of the DOX-loaded M2 micelles was greater than the corresponding blank micelles [22]. The drug-loading efficiency (DLE) and drug-loading content (DLC) of the drug-loaded M2 micelles were calculated to be 81.7% and 9.1%, respectively. The in vitro release of DOX from M2 micelles was evaluated at different pH values (7.4 and 6.4, PBS) under physiological conditions. As shown in Figure 6, the cumulative release of DOX from drug-loaded micelles (M2) was ~35% at pH 7.4 after 48 h. However, the DOX release from the M2 micelles was improved and extended to 56.0% at pH 6.4 after 48 h, which was attributed to the presence of the primary amine group in DOX as expected [43,44]. The primary amine group (pKa = 8.3) of DOX is more protonated at pH 6.4 and thus its solubility in PBS increases, resulting in more favorable release from the polymer micelles. Therefore, the observed increment in DOX release rate at acidic pH (6.4) can be treated as a benefit for an anticancer targeting drug-delivery system.

## 3. Conclusions

In summary, we developed the amphiphilic miktoarm star block copolymers of AB_2_-type containing PLGA hydrophobic polymers and PNAM hydrophilic polymers, synthesized through ROP and RAFT techniques. The size and chemical structure of the miktoarm star block copolymers were analyzed by GPC and ^1^H NMR. The critical micelle concentrations of the miktoarm star block copolymers were determined by DLS. The miktoarm star block copolymers self-assembled to form micelles in water and the resulting micelles were examined by TEM and DLS. DOX was efficiently encapsulated into the micelles with a DLE of 81.7% and DLC of 9.1%. The in vitro drug-release profile of DOX-loaded micelles indicated that as the pH reduces from 7.4 to 6.4, DOX release became faster. The amphiphilic PLGA-b-PNAM_2_ miktoarm star block copolymers may find broad application as nanocarriers in controlled drug delivery.

## 4. Materials and Methods

### 4.1. Materials

Glycolide and D,L-lactide (99.0%, Sigma-Aldrich, Korea) were recrystallized in ethyl acetate. *N*-acryloylmorpholine (98.0%, TCI, Seoul, Korea) was purified by distillation under reduced pressure. Doxorubicin hydrochloride (DOX.HCl) was kindly supplied by Boryung Pharmaceutical Co., Ltd. (Seoul, Korea). AIBN (98%, Sigma-Aldrich, Seoul, Korea), dichloromethane (CH_2_Cl_2_, TCI), tetrahydrofuran (THF, TCI, Korea), benzoic acid (Sigma-Aldrich, Korea), dimethylformamide (DMF, Sigma-Aldrich, Korea) and 1,8-diazabicyclo [5.4.0] undec-7-ene (DBU, 98%) were purchased from Sigma-Aldrich (Korea).

### 4.2. Synthesis of 2-Ethyl-2-(Hydroxyl Methyl) Propane-1, 3-Diyl Bis(2-((Ethoxy Carbonothioyl) Thio) Propanoate) (Miktoarm Initiator)

A detailed methodology for the synthesis of the miktoarm initiator is described in the Supporting Information.

### 4.3. Synthesis of PLGA Copolymer by ROP

The PLGA copolymer was prepared by ROP of D, L-lactide and glycolide using the miktoarm initiator and DBU catalyst. In brief, 1.0 g (6.93 × 10^−2^ mol) of D, L-lactide and 4.0 mL of dry DCM was placed in a 100-mL dry RB flask under nitrogen atmosphere in a glove box. Then, 20 mg (1.20 × 10^−4^ mol) of the miktoarm initiator and 10 μL (10 mg, 3.9 × 10^−5^ mol) of DBU were added to the above RB flask. The reaction mixture was stirred at room temperature. Then, the solution of 0.3 g of glycolide in 2.0 mL of THF was dropwise added to the reaction mixture and the reaction continued for 5 min. Then, the polymerization was stopped by adding 39 mg (3.9 × 10^−5^ mol) of benzoic acid. The obtained copolymer was purified by precipitating in cold methanol (−10 °C). The copolymer was precipitated in methanol twice and finally dried under vacuum at room temperature for 24 h.

^1^H NMR (600 MHz, CDCl_3_): *δ* (ppm) = 5.1–5.25 (1H_b_), 4.88–478 (2Ha), 4.65–4.58 (4H_k_), 4.4–4.3 (2H_d_), 4.30–4.26 (2H_h_), 4.1–4.0 (2H_e_), 1.62–1.20 (3H_c_ + 6H_i_ + 6H_l_), 0.87–0.85 (3H_f_).

*M*_n_(NMR) = 4800 g mol^−1^, *M*_n_(GPC) = 5300 g mol^−1^ and *Đ* = 1.20.

### 4.4. Synthesis of PLGA-b-PNAM_2_ Miktoarm Star Block Copolymers by RAFT Polymerization (M1)

The PLGA-b-PNAM_2_ miktoarm star block copolymers were prepared by the RAFT method. Typically, into a dried reaction flask, 0.1 g (0.020 mmol) of the PLGA macro initiator, 0.15 g (1.04 mmol) of NAM and 0.7 mg (0.010 mmol) of AIBN in 4 mL of dry DMF were added. After purging with N_2_ gas for 30 min, the reaction started at 80 °C with magnetically stirring for 8 h. The polymerization was quenched by exposing it to air in an ice bath. The product was purified by precipitation from cold diethyl ether (−10 °C) and dried in a vacuum oven at 40 °C for 24 h, yielding a white solid (0.22 g, 88%).

^1^H NMR (600 MHz, CDCl_3_):δ (ppm) = 5.25–5.12 (1H_b_), 4.88–478 (2H_a_) 4.65 (4H_k_), 3.9–3.1 (8H_r_), 2.78–2.4 (2H_q_), 1.92–1.65(1H_p_), 1.58–1.20 (3H_c_ + 6H_i_ + 6H_l_), 0.85–0.83 (3H_f_). 

M_n_(NMR) = 10,670 g mol^−1^, M_n_(GPC) = 11,600 g mol^−1^ and Đ = 1.38.

### 4.5. In Vitro Drug Loading and Release Study

Drug loading and drug release of M2 micelles were carried out by following the reported methods [22,43]. In brief, DOX-loaded M2 micelles was geared up by dialysis method. Thirty milligrams of PLGA-b-PNAM_2_ was dissolved in 2.0 mL of DMSO and the mixture was stirred at room temperature for 6 h. Further, 3.0 mg of DOX.HCl was added dropwise, followed by the addition of 3.5 μL of triethylamine. The reaction mixture was left to stir at room temperature for 24 h. Then, the resultant solution was dialyzed (MW cut off = 3500 Da) against of distilled water, which was renewed periodically for every 2 h during the course of initial 15 h, then every 4 h to remove the unloaded drug for 24 h. After the dialysis, dialyzed drug-loaded polymer solution was filtered and lyophilized. In order to determine the DLC and DLE, lyophilized drug-loaded polymer was dissolved in PBS at pH 7.4 and analyzed by UV absorbance at 485 nm, using a standard calibration curve which was experimentally obtained with different concentrations of DOX/PBS (pH = 7.4) solutions. DLC and DLE were calculated according to the following formula:

DLC (wt%) = [Weight of loaded drug/Weight of polymer] × 100

DLE (wt%) = [Weight of loaded drug/Weight in feed] × 100

For the drug-release study, 5.0 mg of the DOX-loaded polymer was dissolved in 1.0 mL of PBS (pH = 7.4) and transferred into a dialysis tube (MW cut off = 3500 Da). Then the tube was placed into 10 mL of PBS. The system was kept under stirring at 37 °C. At a definite interval, 3.0 mL of the PBS solution was taken out, and the solution was replenished with 3 mL of a fresh PBS solution after each sampling. The amount of drug released was estimated by UV spectroscopy at 485 nm.

### 4.6. Characterization

The chemical structure, *M*_n_ and *Đ* of the prepared miktoarm star block copolymers were measured by ^1^H NMR (JEOL, 600 MHz) and GPC (HP 1100 pump, RID detector, PL gel column). The micellar solution of the miktoarm-block copolymer was prepared by dialysis technique. The cmc was determined using a dynamic laser light scattering (Malvern Panalytical’s Zetasizer-1008082, Malvern Panalytical Ltd, Great Malvern, UK). A series of solutions ranging from 1.0 to 1 × 10^−6^ mg/mL was prepared from an aqueous/PBS stock solution of miktopolymer at a concentration of 1% (*w*/*v*). For transmission electron microscopy (TEM; JEOL JEM-2010,Hitachi Ltd., Tokyo, Japan), samples were prepared by dropping micellar solutions on carbon coated copper grids. No staining was applied to the sample.

## Figures and Tables

**Table 1:** Typical results of PLLA–b–(PNAM)_2_ miktoarm star block copolymers **^a^**.

Run	Miktoarm Polymer	NAM ^b^ (Equiv)	Conv. ^c^ (%)	*M*_n_(NMR) ^d^ (g/mol)	Mn(GPC) ^e^ (g/mol)	*Đ*
PLGA	PLGA_26_–macroinitiator	–	88	4800	5300	1.20
M1	PLGA_26_–b–(PNAM_21_)_2_	50	90	10,760	11,600	1.38
M2	PLGA_26_–b–(PNAM_40_)_2_	100	86	16,140	17,400	1.40
M3	PLGA_26_–b–(PNAM_81_)_2_	200	82	25,800	28,000	1.46

**^a^** using 2, 2/-Azobis(isobutyronitrile) (AIBN) initiators (0.5 equivalent) with respect to the poly(D,L-lactide-co-glycolide) (PLGA) macroinitiator in dimethylformamide (DMF) at 80 °C for 8 h; **^b^** with respect to the PLGA macroinitiator.; **^c^** measured gravimetrically; **^d^** calculated by ^1^HNMR; **^e^** measured by gel permeation chromatography (GPC), calibrated against PMMA standards.

## Supplementary Materials

The following are available online at https://www.mdpi.com/1996-1944/13/17/3713/s1, Synthesis of 2-ethyl-2-(hydroxyl methyl) propane-1, 3-diyl bis(2-((ethoxy carbonthioyl) thio) propanoate) (miktoarm initiator, Figure S1: (**a**) Plot of the counter rate versus the concentration of M1, M2 and M3 in water. (**b**) Plot of the micellar size in diameter versus the concentration of M1, M2 and M3 in PBS (pH = 7.4) at 37 °C.

## References

[1] Wang H., Zhao Y., Wu Y., Hu Y.L., Nan K., Nie G., Chen H. (2011). Enhanced anti-tumor efficacy by co-delivery of doxorubicin and paclitaxel with amphiphilic methoxy PEG-PLGA copolymer nanoparticles. Biomaterials.

[2] Zhu Z., Xie C., Liu Q., Zhen X., Zheng X., Wu W., Li R., Ding Y., Jiang X., Liu B. (2011). The effect of hydrophilic chain length and iRGD on drug delivery from poly(epsilon-caprolactone)-poly(N-vinylpyrrolidone) nanoparticles. Biomaterials.

[3] Kamaly N., Yameen B., Wu J., Farokhzad O.C. (2016). Degradable Controlled-Release Polymers and Polymeric Nanoparticles: Mechanisms of Controlling Drug Release. Chem. Rev..

[4] Verma G., Hassan P.A. (2013). Self assembled materials: Design strategies and drug delivery perspectives. Phys. Chem. Chem. Phys..

[5] Khanna K., Varshney S., Kakkar A. (2010). Miktoarm star polymers: Advances in synthesis, self-assembly, and applications. Polym. Chem..

[6] Altintas O., Schulze-Suenninghausen D., Luy B., Barner-Kowollik C. (2015). ABC-type miktoarm star terpolymers accessed by H-bonding driven supramolecular self-assembly. Eur. Polym. J..

[7] Gao H., Matyjaszewski K. (2010). Modular Approaches to Star and Miktoarm Star Polymers by ATRP of Cross-Linkers. Macromol. Symp..

[8] Isono T., Otsuka I., Kondo Y., Halila S., Fort S., Rochas C., Satoh T., Borsali R., Kakuchi T. (2013). Sub-10 nm Nano-Organization in AB2- and AB3-Type Miktoarm Star Copolymers Consisting of Maltoheptaose and Polycaprolactone. Macromolecules.

[9] Babin J., Leroy C., Lecommandoux S., Borsali R., Gnanou Y., Taton D. (2005). Towards an easy access to amphiphilic rod-coil miktoarm star copolymers. Chem. Commun..

[10] Chong Y.K., Zainol I., Ng C.H., Ooi I.H. (2019). Miktoarm star polymers nanocarrier: Synthesis, characterisation, and in-vitro drug release study. J. Polym. Res..

[11] Saravanakumar G., Park H., Kim J., Park D., Pramanick S., Kim D.H., Kim W.J. (2018). Miktoarm Amphiphilic Block Copolymer with Singlet Oxygen-Labile Stereospecific β-Aminoacrylate Junction: Synthesis, Self-Assembly, and Photodynamically Triggered Drug Release. Biomacromolecules.

[12] Bates M.W., Barbon S.M., Levi A.E., Lewis R.M., Beech H.K., Vonk K.M., Zhang C., Fredrickson G.H., Hawker C.J., Bates C.M. (2020). Synthesis and Self-Assembly of ABn Miktoarm Star Polymers. ACS Macro Lett..

[13] Zhu M.-M., Song F., Nie W.-C., Wang X.-L., Wang Y.-Z. (2016). A facile chemoenzymatic synthesis of amphiphilic miktoarm star copolymers from a sugar core and their potential for anticancer drug delivery. Polymer.

[14] Soliman G.M., Redon R., Sharma A., Mejia D., Maysinger D., Kakkar A. (2014). Miktoarm star polymer based multifunctional traceable nanocarriers for efficient delivery of poorly water soluble pharmacological agents. Macromol. Biosci..

[15] Wang M., Zhang X., Peng H., Zhang M., Zhang X., Liu Z., Ma L., Wei H. (2018). Optimization of Amphiphilic Miktoarm Star Copolymers for Anticancer Drug Delivery. ACS Biomater. Sci. Eng..

[16] Peng X., Zhang Y., Chen Y., Li S., He B. (2016). Synthesis and crystallization of well-defined biodegradable miktoarm star PEG-PCL-PLLA copolymer. Mater. Lett..

[17] Yoon K., Kang H.C., Li L., Cho H., Park M.-K., Lee E., Bae Y.H., Huh K.M. (2015). Amphiphilic poly(ethylene glycol)-poly(ε-caprolactone) AB2 miktoarm copolymers for self-assembled nanocarrier systems: Synthesis, characterization, and effects of morphology on antitumor activity. Polym. Chem..

[18] Soliman G.M., Sharma R., Choi A.O., Varshney S.K., Winnik F.M., Kakkar A.K., Maysinger D. (2010). Tailoring the efficacy of nimodipine drug delivery using nanocarriers based on A2B miktoarm star polymers. Biomaterials.

[19] Yin H., Kang S.-W., Bae Y.H. (2009). Polymersome Formation from AB2Type 3-Miktoarm Star Copolymers. Macromolecules.

[20] Erdogan T., Ozyurek Z., Hizal G., Tunca U. (2004). Facile synthesis of AB2-type miktoarm star polymers through the combination of atom transfer radical polymerization and ring-opening polymerization. J. Polym. Sci. Part A Polym. Chem..

[21] Shi Y., Fu Z., Li B., Zhang L., Cai X., Zhang D. (2007). Synthesis of AB2 miktoarm star-shaped copolymers by combining stable free radical polymerization and atom transfer radical polymerization. Eur. Polym. J..

[22] Ramesh K., Thangagiri B., Mishra A.K., Ahn B.-H., Gal Y.-S., Lim K.T. (2018). AB2-type miktoarm poly(l-lactide)-b-poly(N-acryloylmorpholine) amphiphilic star block copolymers as nanocarriers for drug delivery. React. Funct. Polym..

[23] Sun X., Xu C., Wu G., Ye Q., Wang C. (2017). Poly(Lactic-co-Glycolic Acid): Applications and Future Prospects for Periodontal Tissue Regeneration. Polymers.

[24] Ramesh K., Singh S., Mitra K., Chattopadhyay D., Misra N., Ray B. (2016). Self-assembly of Novel Poly(d,l-Lactide-co-Glycolide)-b-Poly(N-Vinylpyrrolidone) (PLGA-b-PNVP) Amphiphilic Diblock Copolymers. Colloid Polym. Sci..

[25] Makadia H.K., Siegel S.J. (2011). Poly Lactic-co-Glycolic Acid (PLGA) as Biodegradable Controlled Drug Delivery Carrier. Polymers.

[26] Wang J., Li S., Chen T., Xian W., Zhang H., Wu L., Zhu W., Zeng Q. (2019). Nanoscale cationic micelles of amphiphilic copolymers based on star-shaped PLGA and PEI cross-linked PEG for protein delivery application. J. Mater. Sci. Mater. Med..

[27] Shen H., Niu Y., Hu X., Yang F., Wang S., Wu D. (2015). A biomimetic 3D microtubule-orientated poly(lactide-co-glycolide) scaffold with interconnected pores for tissue engineering. J. Mater. Chem. B.

[28] Chaduc I., Reynaud E., Dumas L., Albertin L., D’Agosto F., Lansalot M. (2016). From well-defined poly( N -acryloylmorpholine)-stabilized nanospheres to uniform mannuronan- and guluronan-decorated nanoparticles by RAFT polymerization-induced self-assembly. Polymer.

[29] Rivas B.L., Maureira A., Geckeler K.E. (2006). Novel water-soluble acryloylmorpholine copolymers: Synthesis, characterization, and metal ion binding properties. J. Appl. Polym. Sci..

[30] Savelyeva X., Lessard B.H., Marić M. (2012). Amphiphilic Poly(4-acryloylmorpholine)/Poly[2-(N-carbazolyl)ethyl acrylate] Random and Block Copolymers Synthesized by NMP. Macromol. React. Eng..

[31] Fares M.M., Al-Shboul A.M. (2012). Stimuli pH-responsive (N-vinyl imidazole-co-acryloylmorpholine) hydrogels; mesoporous and nanoporous scaffolds. J. Biomed. Mater. Res. Part A.

[32] Xu F., Li H., Luo Y.L., Tang W. (2017). Redox-Responsive Self-Assembly Micelles from Poly(N-acryloylmorpholine-block-2-acryloyloxyethyl ferrocenecarboxylate) Amphiphilic Block Copolymers as Drug Release Carriers. ACS Appl. Mater. Interfaces.

[33] Samarajeewa S., Shrestha R., Li Y., Wooley K.L. (2012). Degradability of poly(lactic acid)-containing nanoparticles: Enzymatic access through a cross-linked shell barrier. J. Am. Chem. Soc..

[34] Xu R., Feng Q., He Y., Yan F., Chen L., Zhao Y. (2017). Dual functionalized poly(vinylidene fluoride) membrane with acryloylmorpholine and argatroban to improve antifouling and hemocompatibility. J. Biomed. Mater. Res. Part A.

[35] Takahashi H., Nakayama M., Itoga K., Yamato M., Okano T. (2011). Micropatterned thermoresponsive polymer brush surfaces for fabricating cell sheets with well-controlled orientational structures. Biomacromolecules.

[36] Chado G.R., Holland E.N., Tice A.K., Stoykovich M.P., Kaar J.L. (2018). Modification of Lipase with Poly(4-acryloylmorpholine) Enhances Solubility and Transesterification Activity in Anhydrous Ionic Liquids. Biomacromolecules.

[37] Ramesh K., Mishra A.K., Patel V.K., Vishwakarma N.K., Biswas C.S., Paira T.K., Mandal T.K., Maiti P., Misra N., Ray B. (2012). Synthesis of well-defined amphiphilic poly(d,l-lactide)-b-poly(N-vinylpyrrolidone) block copolymers using ROP and xanthate-mediated RAFT polymerization. Polymer.

[38] Mayadunne R.T.A., Jeffery J., Moad G., Rizzardo E. (2003). Living Free Radical Polymerization with Reversible Addition−Fragmentation Chain Transfer (RAFT Polymerization):  Approaches to Star Polymers. Macromolecules.

[39] Boschmann D., Vana P. (2007). Z-RAFT star polymerizations of acrylates: Star coupling via intermolecular chain transfer to polymer. Macromolecules.

[40] Lu J., Owen S.C., Shoichet M.S. (2011). Stability of Self-Assembled Polymeric Micelles in Serum. Macromolecules.

[41] Jhaveri A.M., Torchilin V.P. (2014). Multifunctional polymeric micelles for delivery of drugs and siRNA. Front. Pharmacol..

[42] Elsabahy M., Wooley K.L. (2012). Design of polymeric nanoparticles for biomedical delivery applications. Chem. Soc. Rev..

[43] Ramesh K., Anugrah D.S.B., Lim K.T. (2018). Supramolecular poly(N-acryloylmorpholine)-b-poly(d,l-lactide) pseudo-block copolymer via host-guest interaction for drug delivery. React. Funct. Polym..

[44] Sanson C., Schatz C., Le Meins J.-F., Soum A., Thévenot J., Garanger E., Lecommandoux S. (2010). A simple method to achieve high doxorubicin loading in biodegradable polymersomes. J. Control. Release.

