# Supplementary material for: Preparation of Doxorubicin-Loaded Amphiphilic Poly(D,L-Lactide-Co-Glycolide)-b-Poly(N-Acryloylmorpholine) AB2 Miktoarm Star Block Copolymers for Anticancer Drug Delivery
Source: Materials (Basel). 2020 Aug 22;13(17):3713. doi: 10.3390/ma13173713 (PMC7504487; doi:10.3390/ma13173713)
Supplement: Supplementary file 1 [file materials-13-03713-s001.pdf]

materials

Supplementary Materials: Preparation of Doxorubicin-Loaded Amphiphilic Poly(D,L-Lactide-*Co*-Glycolide)-b-Poly(*N*-Acryloylmorpholine) AB$_2$ Miktoarm Star Block Copolymers for Anticancer Drug Delivery

Kalyan Ramesh, Avnish Kumar Mishra, Jin Kon Kim, Yeon Tae Jeong, Yeong-Soon Gal and Kwon Taek Lim

Synthesis of 2-ethyl-2-(hydroxyl methyl) propane-1, 3-diyl bis(2-((ethoxy carbonthioyl)thio)propanoate) (miktoarm initiator)

The miktoarm initiator, 2-Ethyl-2-(hydroxyl methyl)propane-1, 3-diyl bis(2-((ethoxycarbonthioyl)thio)propanoate) was prepared by previous reported method [1].

First, 2-ethyl-2-(hydroxymethyl) propane-1,3-diyl bis(2-bromopropanoate) was prepared as below. Pyridine (1.5 mL, 18.66 mmol) was added to 2-ethyl-2-(hydroxymethyl) propane-1,3-diol (1.25 g, 9.33 mmol) in THF (25 mL). The reaction mixture was cooled in an ice-water bath, and 2-bromopropionyl bromide (4 g, 18.66 mmol) in THF (30 mL) was slowly added to the mixture. The mixture was continuously stirred in the ice bath for 1 h and then at room temperature for 16 h. The reaction mixture was then poured into acidic water (pH = 2, 100 mL) and extracted with dichloromethane (3 × 60 mL). The organic fractions were combined, extracted with water, dried on $MgSO_4$ and evaporated. The product was purified by column chromatography and dried under vacuum at room temperature.

Next, a 50 mL, round-bottomed flask was charged with potassium ethyl xanthogenate (0.87 g, 5.44 mmol) and acetone (15 mL). A solution of 2-ethyl-2-(hydroxyl methyl) propane-1,3-diyl bis(2-bromopropanoate) (1 g, 2.47 mmol) in acetone (15 mL) was added dropwise into the flask at room temperature over a period of 30 min. The reaction was continued for 12 h at room temperature. Solids were removed by filtration to give a pale-yellow solution. The solids were washed with 50 mL of acetone. The combined washing and filtrate solutions were concentrated under vacuum to furnish a yellow viscous liquid. The viscous liquid was dissolved in 100 mL of dichloromethane and the solution was washed with water (75 mL). The solution was dried over MgSO4, evaporated and dried under vacuum. Finally, 1.1 g of a clear yellow oil (yield: 91.6 %) product was obtained.

^{1}H NMR (600 MHz, DMSO-d$_6$, ppm): 4.7 (s, 1H$_a$), 4.6 (q, J = 7.1 Hz, 4H$_h$), 4.30 (q, J = 7.3 Hz, 2H$_f$), 3.3 (s, 2H$_b$), 1.45 (d, , J = 7.40 Hz, 3H$_g$), 1.35-1.30 (m, 2H$_c$ + 6Hi), 0.86 (t, J = 7.60 Hz, 3H$_e$).

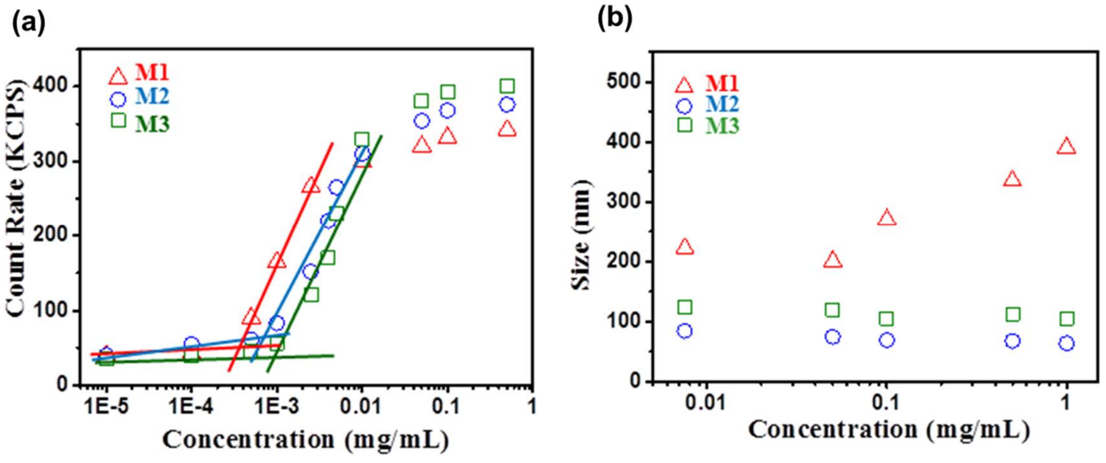

Figure S1. (**a**) Plot of the counter rate versus the concentration of M1, M2 and M3 in water. (**b**) Plot of the micellar size in diameter versus the concentration of M1, M2 and M3 in PBS (pH = 7.4) at 37 °C.

References

1. Ramesh, K.; Thangagiri, B.; Mishra, A.K.; Ahn, B.-H.; Gal, Y.-S.; Lim, K.T. AB2-type miktoarm poly(l-lactide)-b-poly(N-acryloylmorpholine) amphiphilic star block copolymers as nanocarriers for drug delivery. Reactive and Functional Polymers 2018, 132, 112-119, doi:https://doi.org/10.1016/j.reactfunctpolym.2018.09.019.

© 2020 by the authors. Submitted for possible open access publication under the terms and conditions of the Creative Commons Attribution (CC BY) license (http://creativecommons.org/licenses/by/4.0/).
